# Spatial organization of enzymes to enhance synthetic pathways in microbial chassis: a systematic review

**DOI:** 10.1186/s12934-018-0965-0

**Published:** 2018-07-31

**Authors:** Xin-yuan Qiu, Si-Si Xie, Lu Min, Xiao-Min Wu, Lv-Yun Zhu, Lingyun Zhu

**Affiliations:** Department of Biology and Chemistry, College of Liberal Arts and Sciences, National University of Defense Technology, Changsha, 410073 Hunan China

**Keywords:** Spatial organization, Enzyme clustering, Pathway enhancement, Molecular scaffold

## Abstract

For years, microbes have been widely applied as chassis in the construction of synthetic metabolic pathways. However, the lack of in vivo enzyme clustering of heterologous metabolic pathways in these organisms often results in low local concentrations of enzymes and substrates, leading to a low productive efficacy. In recent years, multiple methods have been applied to the construction of small metabolic clusters by spatial organization of heterologous metabolic enzymes. These methods mainly focused on using engineered molecules to bring the enzymes into close proximity via different interaction mechanisms among proteins and nucleotides and have been applied in various heterologous pathways with different degrees of success while facing numerous challenges. In this paper, we mainly reviewed some of those notable advances in designing and creating approaches to achieve spatial organization using different intermolecular interactions. Current challenges and future aspects in the further application of such approaches are also discussed in this paper.

## Background

With the rapid development of molecular biology, genomics and bioengineering, synthetic biology, as a new interdisciplinary area of biology and engineering, has been developing rapidly [1]. By integrating cascade enzymes into self-replicable microbes, microbial cell factories (MCFs), as a new concept in synthetic biology, provide a brand-new approach and a revolutionary platform for the biosynthesis of multiple products. Among all those chassis being used, prokaryotic cells are widely applied as host cells due to their rapid growth and higher productivity over eukaryotic cells [2]. Recently, prokaryotic MCFs have been widely used to produce many chemicals. For example, *Corynebacterium glutamicum* was used in the biosynthesis of medical materials such as l-amino acids [3–6], chemicals such as 1,5-butanediamine [7] and 1,4-butanediamine [8], as well as bioenergy substances such as ethanol [9]. *Escherichia coli*, as another important chassis, was also widely used in similar fields, where some of those applications were even widely applied in industrial production [10–13]. Notably, the production of terpenoids such as lycopene and α-pinene in MCFs was achieved by modifying the biosynthesis pathways of terpenoids in *E. coli* [14], which recently showed the potential of MCFs in the production of highly important and high-value products.

**Fig. 1:**
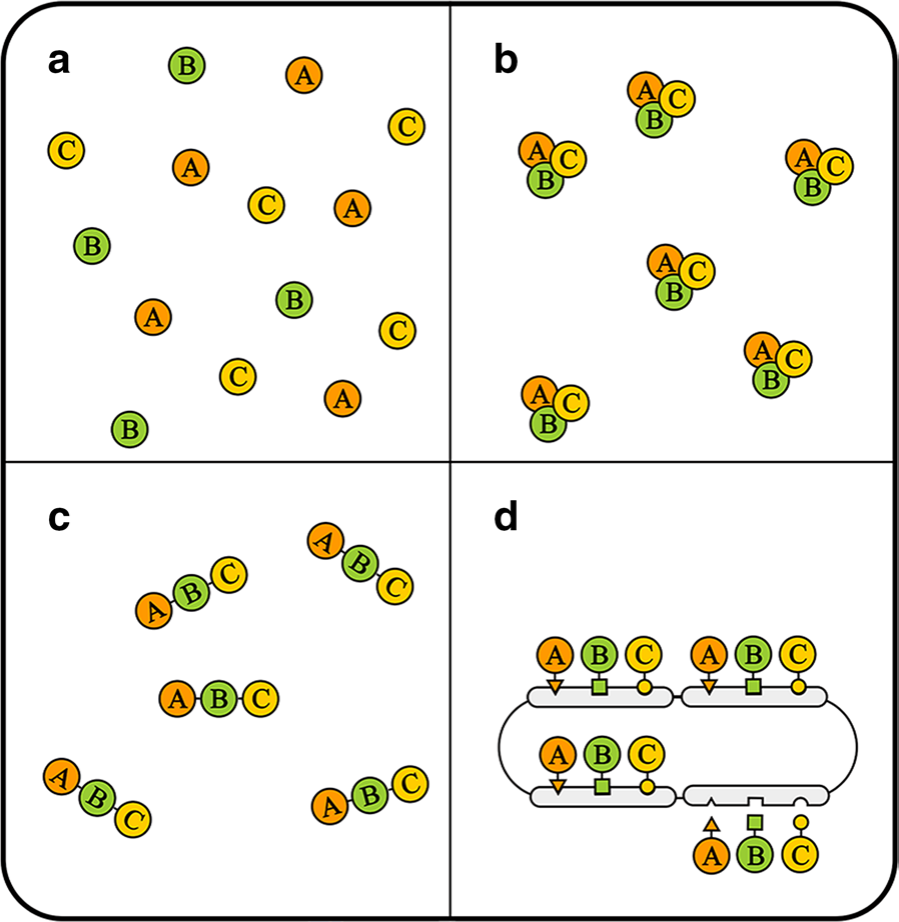
Different modes of enzyme clustering in vivo. **a** Enzymes without specific spatial organization. **b** Enzyme clusters in natural metabolic pathways. Enzyme can form complexes with specific spatial structure that is capable of enhancing metabolic pathways. **c** Traditional direct fusion method clustering pathway enzymes. Enzymes are fused from end to end, forming a synthetic enzyme complex. **d** Clustering mode based on the interaction among biomolecules. Enzymes fused with specific adaptor can be recruited by medium molecules forming a metabolic microdomain

With the increased complexity of the MCFs, the collision between the production of target products and the survival of chassis themselves have been highlighted in multiple studies. Such collision, observed as crosstalks among pathways, degradation of important intermediates and enrichment of toxic by-products, is mainly due to the weak multienzyme colocalization mechanisms in prokaryotes [15–17] (Fig. 1a). Its existence severely impacts the efficiency of production and efficacy of target products [18].

To address this issue, inspired by the naturally existing, pathway-enhancing compartments or molecular clusters (Fig. 1b), multiple approaches were implemented to achieve the spatial organization of heterologous metabolic pathways [19, 20]. Through this way, the concentration of enzymes, substrates and intermediates can be enriched locally, and the crosstalk among pathways can thus be reduced.

Early attempts for spatial organization of pathway enzymes mainly focused on the direct fusion of those enzymes (Fig. 1c). In 2011, Wang et al. [21] reported a novel approach to enhance the α-farnesene synthetic pathway in *E. coli*. Meynial et al. [22] also reported a method to enhance a metabolic pathway in *S. cerevisiae* by fusing the pathway enzymes together in 2017. These two studies are the representative applications of enzyme-fusion in pathway enhancement. However, upon further examination of enzyme-fusion approaches, the negative influence of these methods on the structure and correct folding of enzymes was gradually noted, thus severely limiting the application of such strategy on pathways containing more than three enzymes [23].

Hence, more researchers have started to find other approaches to achieve the spatial organization of pathway enzymes. Inspired by the interaction among biomolecules, several scaffold systems, mainly based on the interaction among proteins and nucleotides, were established these years (Fig. 1d) and present great advantages and remarkable pathway-enhancing abilities compared to previous approaches [23].

In this paper, we mainly discuss some of those notable advances in designing and creating approaches to achieve spatial organization using different intermolecular interactions. We also discuss the application of genome-editing technologies on developing new spatial organization systems. The current challenges and future aspects in further applications of such approaches are also highlighted and discussed in this paper.

## Mediating the spatial organization of enzymes: from protein to nucleotide

To achieve the clustering of exogenous enzymes, multiple interaction mechanisms have been applied to improve the proximity of those enzymes. Among them, protein–protein interaction, nucleotide base-pairing, and aptamer-adaptor interactions, as the most well-characterized inter-molecular interactions, have been widely used to enhance exogenous pathways.

**Fig. 2:**
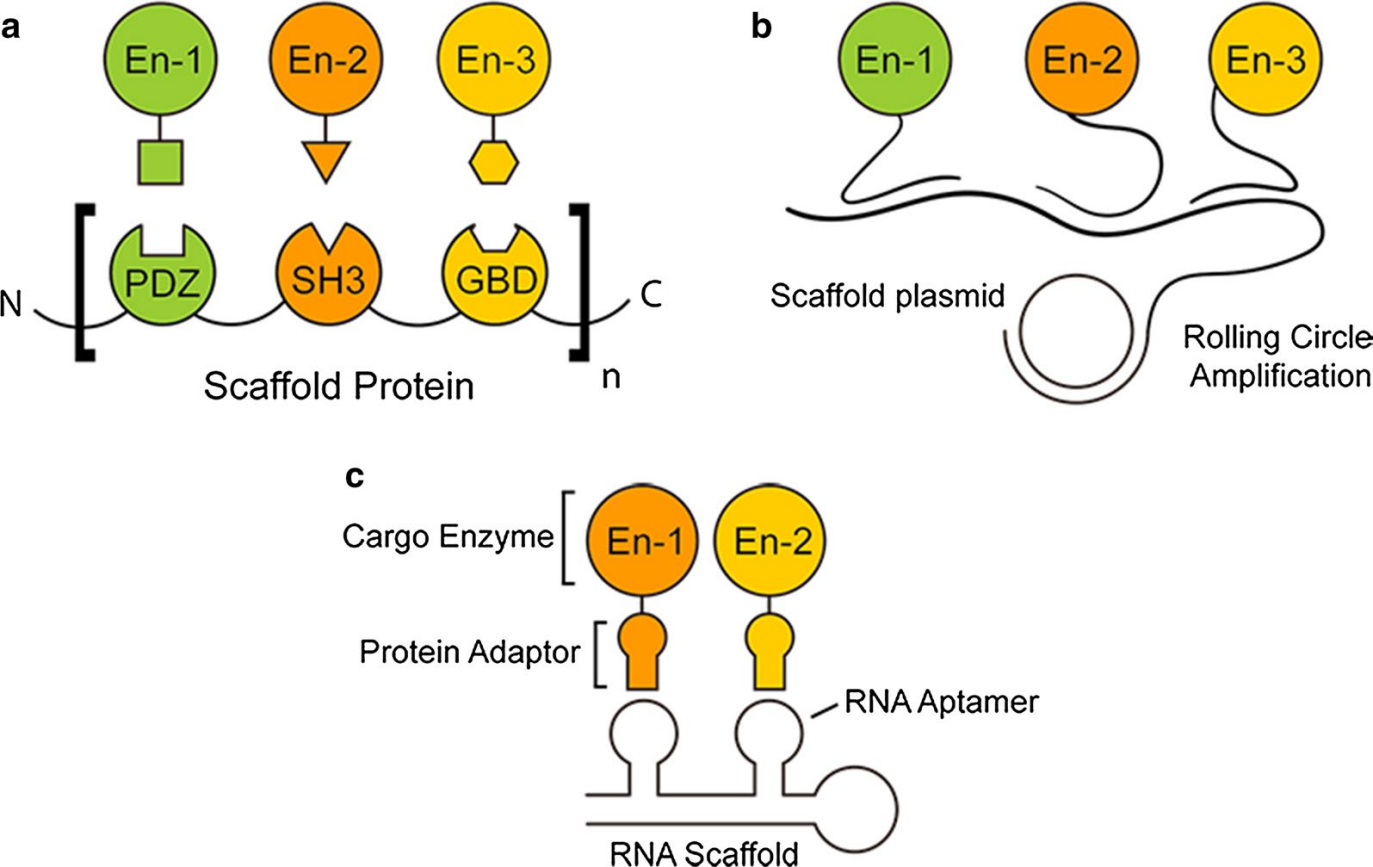
Typical protein or nucleotide based scaffolding approaches. **a** Typical approach based on the protein–protein interactions. SH3, PDZ and GBD domains are modified as medium molecule, while their corresponding ligands are fused with pathway enzymes. **b** Typical approach based on the nucleotide-nucleotide interactions. Single strand DNA produced from phi29 DNA polymerase through rolling circle amplification is used as medium molecule, while its complementary strands are chemically bond to the pathway enzyme. **c** Typical approach based on the aptamer-adaptor interactions. Synthesized RNA strand with specific secondary structure known as aptamers are used as medium molecule, while pathway enzymes are fused with adaptor proteins

Performing important biochemical functions, protein–protein interactions exist widely in cells or living organisms under specific biomolecular context. In recent years, complex protein interaction networks as well as detailed structures of multiple protein binding regions have been discovered and well characterized. With these new findings, in 2009, Dueber et al. [24] used the interaction between SH3, PDZ and GBD domains and their corresponding ligands to construct multi-enzymatic complexes, thus improving the performance of the heterologous biosynthesis of mevalonate in *E. coli* (Fig. 2a). With some further optimization, the production of mevalonate increased by ~ 77-fold compared to the control system. It is interesting to find that tremendously high-yield improvement only occurred on specific occasions, while other scaffolds, though with similar design, showed only a limited pathway-enhancing effect. i.e., in another study that used the same domains to increase the production of glucaric acid in *E. coli*, the production titer of glucaric acid was improved by only fivefold compared to the control group [25]. Protein-based systems have also been used in vitro to generate metabolons using the surface of the microbes as a platform by implementing a surface-display technique. Liu et al. [26] exploited an approach using the cohesin–dockerin interactions to assemble protein complexes containing three NAD^+^-dependent dehydrogenases on the surface of yeast, and via that approach, the NADH production was improved by 5 times over the control. Similarly, Tsai et al. [27] developed a *S. cerevisiae* surface-display system to produce ethanol in vitro. The production titer of such method was reported to be three to fourfold higher than that of the control group. The proliferating cell nuclear antigen (PCNA) from *S. solfataricus* and some other cyclic protein domains have also been reported to be used as a protein base to achieve the spatial organization of specific enzymes in vitro [28–30].

With the rapid development of nucleotide-modification technology, nucleotide-nucleotide interaction is also used for the spatial organization of pathway enzymes. Since the structure of DNA and RNA, as well as the base-pairing mechanisms, have been well-understood, the design and construction of nucleotide-nucleotide interaction-based enzyme clusters are thought to be more predictable and applicable compared to the protein–protein interaction strategy [31, 32]. An early attempt to demonstrate nucleic acid as a feasible medium for spatial organization of pathway enzymes was reported by Wilner et al. [33] in 2009. In this study, they successfully mounted chemically modified glucose oxidase and horseradish peroxidase (with single-strand DNAs fused on their N-terminal) onto a scaffold-functioning single-strand DNA, which was produced through a rolling circle amplification process (Fig. 2b). This design was then expanded into a two-dimensional multiple-hexagon DNA structure. Significant pathway enhancing effect was observed in their design, as well as another research group using DNA osrigami to build three-dimensional structures for enzyme clustering [33].

Additionally, with a well-characterized structure and function, RNA aptamer motifs have been widely modified to bind adaptor domain fused enzymes. Through the highly specific and strong binding interaction between RNA aptamers and their corresponding protein adaptors, the single-strand RNA containing enough aptamer motifs can be used as a medium structure to achieve the spatial organization of adaptor-fused enzymes (Fig. 2c). Using this approach, in 2011, Delebecque et al. [34] reported an RNA aptamer-based system that increased hydrogen production efficiency by up to 48-fold through the ferredoxin-[Fe–Fe] hydrogenase pathway. The primary sequence of these RNAs can be rationally designed to control both the distance and orientation between bound proteins, as well as their stoichiometry and the size of the overall complexes. A set of designing principles and workflow were then established [35]. Building on these works, Schdeva et al. [36] reported a complex two-dimensional RNA structure that can be employed in living *E. coli*, which showed significant enzyme clustering effect and improved the productivity of a succinate synthesis pathway by 88%.

Although previous applications of different metabolic pathways somehow proved the effectiveness of these systems, challenges remain, especially with their robustness and scalability. For example, protein-based spatial organization systems mainly rely on specific protein interaction domains, whose detailed mechanisms are still yet to be determined. Additionally, the increase of pathway enzymes will undoubtedly increase the molecular size of the medium protein, thus dramatically increasing the difficulty of protein expression and folding. For DNA-based spatial organization systems, the chemical modification of DNA-fused enzymes is highly costly and extremely unstable in vivo, since single-strand DNA structures can be easily degraded by DNase in the cytoplasm.

## Genome-editing techniques: new hints for developing spatial organization systems

Since the establishment of novel artificial spatial organization methods should meet the requirements of natural, highly stable, expandable, and simple to be applied in various metabolic systems and chassis. Recently developed genome-editing tools [e.g., zinc finger nucleases (ZFN), transcription activator-like effector nuclease (TALEN), and Clustered regularly interspaced short palindromic repeats and its associated protein 9 (CRISPR-Cas9)] can precisely meet these requirements and provide clues for new spatial organization systems. To precisely edit genome, genome editing tools often rely on highly effective and sequence-specific DNA-binding. For example, zinc finger nuclease contains a engineerable zinc finger domain, which consists of three to six replaceable individual zinc finger repeats to bind desired DNA sequence [37]. Similarly, TALENs share similar TALE domains that contain a certain amount (approximately 1.5–33.5) of TAL repeats, each of which are able to recognize a specific DNA base pair [38]. These DNA-binding domains allow site-specific cutting directed by their fused nuclease domains, thus demonstrating the possibility that the exact DNA-binding domains may be used to spatially organize metabolic enzymes around a DNA scaffold. Additionally, the rapid developing CRISPR-Cas9 technique, which is mediated by a single guide RNA (sgRNA) and Cas9 nuclease [39], also provided a promising approach for developing DNA scaffold-based, higher dimensional enzyme clustering systems.

**Fig. 3:**
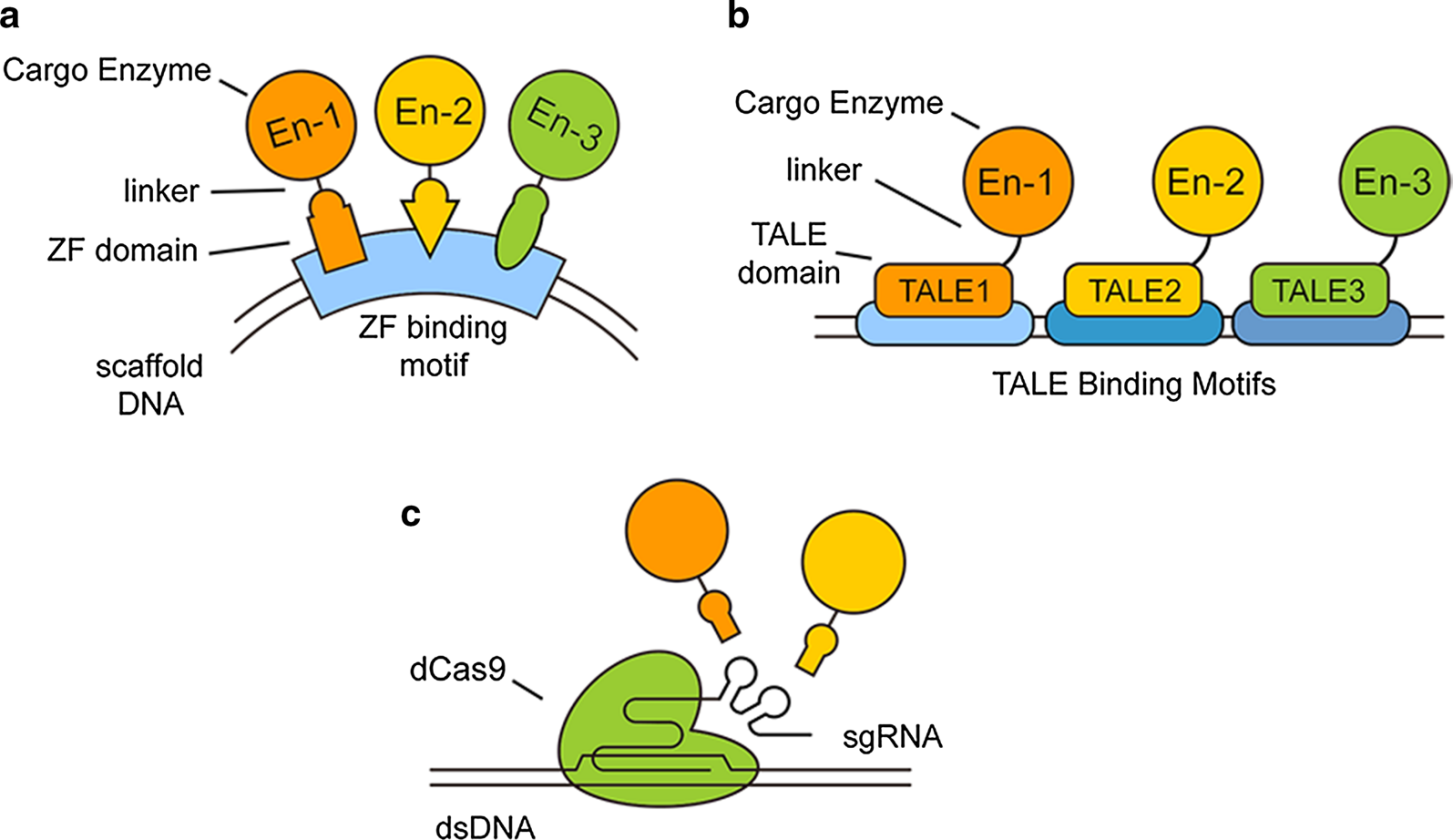
Typical scaffolding approaches based on genome editing tools. **a** ZFP-based method using synthesized plasmid DNA containing binding motifs of ZFPs as medium molecule, enzymes are fused with zinc finger domains. **b** TALE based approach using DNA containing multiple binding motifs of TALEs as medium molecule. Enzymes are fused with artificial designed and constructed TALE domains with high DNA-binding affinity and specificity. **c** CRISPR-Cas9 system and its potential on spatial organization of enzymes

Accordingly, recent studies have developed multiple enzyme clustering approaches with these DNA-binding proteins considering their potential for scalability and robustness. Currently, approaches inspired by ZFN and TALEN have already been used for the spatial organization of exogenous enzymes. In 2012, Conrado et al. [40] reported a zinc finger-based system using DNA as scaffold medium to improve catalytic efficiency (Fig. 3a), indicating a powerful DNA-based tool in vivo on the basis of the specific and direct combination between DNA and proteins. In 2016, we demonstrated a TALE-based system for the spatial organization of heterologous metabolic system in vivo. In our assay, pathway enzymes were directly fused with the modified synthetic TALE domain, which was designed to recognize and bind specific DNA sequences (Fig. 3b). Using plasmid DNA as a scaffold, enzymes were proved to be colocalized around the plasmid. An approximately tenfold improvement on the productivity of an indole-3-acetic synthetic pathway in *E. coli* was also reported [41]. Compared to the ZFN approach, the TALEN technique provides a more efficient and practical approach for the clustering of multienzymes in vivo given its simpler design, higher specificity and lower toxicity [42]. Using the same strategy, the production of mevalonate was also improved by ~ 3.7-fold in our follow-up study (unpublished).

More interestingly, a recent study applied the CRISPER–Cas9 technique as a novel system to concentrate specific enzymes for metaplastic functions [43]. In this method, the single guide RNA (sgRNA) was modified to add an aptamer motif on its 3′ end. Due to the RNA-guided DNA-binding ability of dead Cas9 (dCas9) protein, the modified sgRNA can thus be anchored to a DNA structure. In this study, transcriptional regulators fused with the adaptor motif were proven to be clustered around the dCas9 protein (Fig. 3c). Similarly, other studies also tried to fuse the GCN4 scaffold protein with the dCas9 protein, recruiting scFv fused transcriptional activators [44]. The success of these studies in reprogramming transcriptional circuits implies that enzymes fused with corresponding adaptors can also be clustered in a similar manner. However, this potential has not been experimentally validated.

As a new and rapidly advancing strategy for the spatial organization of pathway enzymes, the DNA-binding mechanism in genome editing tools provides a promising way to establish highly stable, extendable, and low-cost systems for such applications. On the other hand, the development of such methods also provides a new vision on how techniques such as genome editing could be used besides its current application in genome editing. In the meanwhile, it should be noted that, with further study on the structural basis and mechanisms of protein-nucleotide interactions, current DNA-binding domains can be further engineered to generate more robust DNA-binding proteins for new spatial organization systems in the near future.

## Conclusion

Since the metabolic pathways are basically a set of enzymes with different dynamics, finding ways for balancing the metabolic flow and reducing negative impact to the chassis is now an important field for synthetic biologists. Despite the strategies employed, the purpose of the spatial control and organization of pathway enzymes remains the same. As an important complementary and parallel approach along with traditional ways, such as rational design of expression systems and optimization of enzyme expression levels, the spatial organization of pathway enzymes aims to provide a highly predictable and stable control of the heterogeneous metabolic pathways and reduce the effect on the survival and growth of the chassis.

However, since the detailed reason why spatial organization of pathway enzymes can enhance the heterologous pathways is yet to be understood, improving the predictability and stability of such scaffolds is still a challenge. Supported by both computational modeling and wet-lab experiments [45], the metabolite microdomain hypothesis claims that the probability of intermediates being processed by downstream enzymes in these enzyme-clustering microdomains is much higher than the free-standing condition, thus providing theoretical foundation for enhancing heterologous pathways through spatial organization of pathway enzymes. However, details on structural and biochemical factors on the pathway enhancing effect of this organization is still to be defined, a biochemical or mathematical tool based on which the scaffold systems can be designed rationally is still in scarce need.

In addition, according to either numerical experiments or wet-lab results currently reported, the benefits of the spatial organization of pathway enzymes are affected by multiple factors such as spatial orientation of enzymes, distance between enzymes, and copy number of scaffold molecules. With multiple, convoluted unknown variables, the rational design and functional examination of scaffold systems are difficult. Hence, further study on structural biology, especially on the detailed mechanisms of the pathway-enhancing effect of spatial organization, is needed. An in-depth investigation on the substrate channeling process on natural existing enzyme clusters, such as the tryptophan synthesis complex, would be a great start. Knowledge on the influencing mechanisms of these factors will provide a sound foundation for the design and creation of new spatial organization methods, especially for highly tunable protein-nucleotide interaction strategies. Moreover, the rapid development and uncovering of new genome editing techniques such as the CRISPR-C2c2 [46] and CRISPR-Cpf1 [47] systems may provide new protein-nucleotide recognition and binding approaches for the development of new spatial organization systems.

## Abbreviations

MCFs: microbial cell factories
SH3 domain: SRC homology 3 domain
GBD domain: GTPase-binding domain
NAD^+^: oxidized form of nicotinamide adenine dinucleotide
NADH: reduced form of nicotinamide adenine dinucleotide
PCNA: the proliferating cell nuclear antigen
CRISPR: clustered regularly interspaced short palindromic repeats
TALEN: transcription activator-like effector nuclease
ZFN: zinc-finger nucleases
sgRNA: single guide RNA
